# *Histoplasma capsulatum*-Induced Cytokine Secretion in Lung Epithelial Cells Is Dependent on Host Integrins, Src-Family Kinase Activation, and Membrane Raft Recruitment

**DOI:** 10.3389/fmicb.2016.00580

**Published:** 2016-04-22

**Authors:** Paloma K. Maza, Erika Suzuki

**Affiliations:** Department of Microbiology, Immunology and Parasitology, Escola Paulista de Medicina, Universidade Federal de São PauloSão Paulo, Brazil

**Keywords:** *Histoplasma capsulatum*, fungi, epithelial cells, cytokine, integrin, membrane rafts, Src-family kinases

## Abstract

*Histoplasma capsulatum* var. *capsulatum* is a dimorphic fungus that causes histoplasmosis, a human systemic mycosis with worldwide distribution. In the present work, we demonstrate that *H. capsulatum* yeasts are able to induce cytokine secretion by the human lung epithelial cell line A549 in integrin- and Src-family kinase (SFK)-dependent manners. This conclusion is supported by small interfering RNA (siRNA) directed to α3 and α5 integrins, and PP2, an inhibitor of SFK activation. siRNA and PP2 reduced IL-6 and IL-8 secretion in *H. capsulatum*-infected A549 cell cultures. In addition, α3 and α5 integrins from A549 cells were capable of associating with *H. capsulatum* yeasts, and this fungus promotes recruitment of these integrins and SFKs to A549 cell membrane rafts. Corroborating this finding, membrane raft disruption with the cholesterol-chelator methyl-β-cyclodextrin reduced the levels of integrins and SFKs in these cell membrane domains. Finally, pretreatment of A549 cells with the cholesterol-binding compound, and also a membrane raft disruptor, filipin, significantly reduced IL-6 and IL-8 levels in A549-*H.capsulatum* cultures. Taken together, these results indicate that *H. capsulatum* yeasts induce secretion of IL-6 and IL-8 in human lung epithelial cells by interacting with α3 and α5 integrins, recruiting these integrins to membrane rafts, and promoting SFK activation.

## Introduction

Histoplasmosis is a human systemic mycosis caused by the fungal pathogen *Histoplasma capsulatum*. This fungus presents two varieties: *H. capsulatum* var. *capsulatum* and *H. capsulatum* var. *duboisii* that are etiological agents of the classical and the African histoplasmoses, respectively. Classical histoplasmosis is widely distributed in the Americas. In the United States, highly endemic areas include the Mississippi and Ohio River valleys. This mycosis also occurs in countries of Central and South America, and in Brazil, outbreaks of histoplasmosis have been reported after exposure to *H. capsulatum* fragments (Martins et al., 2003; Guimarães et al., 2006; Oliveira et al., 2006; Rocha-Silva et al., 2014).

*Histoplasma capsulatum* is a thermally dimorphic fungus, which is found in soil, caves, and abandoned constructions that are enriched in bat or bird excrements (Smith and Kauffman, 2012). Infection with *H. capsulatum* occurs by inhaling microconidia or mycelial fragments which then settle in the host’s lungs and convert to yeast forms (Mihu and Nosanchuk, 2012). Some fungi are internalized by phagocytes and are able to survive and multiply within macrophages, allowing, in this manner, dissemination of *H. capsulatum* to several organs through the bloodstream or lymphatic system (Mihu and Nosanchuk, 2012; Adenis et al., 2014).

The severity of this mycosis depends on the number of inhaled fungal particles and the immune status of the host. In immunocompetent individuals, a small inoculum can cause asymptomatic infection or acute pulmonary histoplasmosis. Individuals with pre-existing lung diseases, such as emphysema, may develop chronic pulmonary histoplasmosis, and immunocompromised patients may present disseminated histoplasmosis (Smith and Kauffman, 2012). Histoplasmosis is responsible for low rates of morbidity and mortality among immunocompetent patients. However, among immunocompromised patients, morbidity, and mortality of this mycosis have increased mostly due to HIV (Adenis et al., 2014). Histoplasmosis is an AIDS-defining illness, and some authors consider HIV-associated histoplasmosis a neglected disease in South America. Unhappily, these cases are often confused with tuberculosis or pneumocystosis (Nacher et al., 2013). In Brazil, in the state of Ceará, a study of a 4-years period (2006–2010) reported 208 cases of histoplasmosis in HIV-positive patients. Histoplasmosis was the first indicator of AIDS in about 39% of the cases. About 80% of these patients were not being treated with highly active antiretroviral therapy (HAART) at the moment of histoplasmosis diagnosis, and about 42% of these patients died (Brilhante et al., 2012).

Besides acting as a structural barrier, several research groups have demonstrated the importance of epithelial cells in modulating the immune system in various body tissues. In the lungs, for example, type II pneumocytes are among the cells that form the alveolar epithelium, and they are involved not only in surfactant production and repair of alveoli after a lung injury, but also in the immune response against particles and inhaled microorganisms (Mason, 2006). To participate in the host’s innate immunity, airway epithelial cells produce a wide range of inflammatory mediators, such as growth factors, cytokines, and chemokines, that promote recruitment and activation of immune cells to the sites of infection (Suzuki et al., 2008; Proud and Leigh, 2011).

Recently, our group has demonstrated that the human fungal pathogen *Paracoccidioides brasiliensis* induces interleukin (IL)-6 and IL-8 secretion by the human lung epithelial cell line A549. This cytokine secretion was dependent on activation of some host cell signaling kinases, such as ERK 1/2 (extracellular signal-regulated kinase 1/2), p38 MAPK (p38 mitogen-activated protein kinase) and PKC δ (protein kinase C δ; Maza et al., 2012; Alcantara et al., 2015). Later, we demonstrated that integrins are one type of receptor involved in the secretion of IL-6 and IL-8 (Barros et al., 2016). In addition, *P. brasiliensis* promoted an increase of integrin expression in these epithelial cells, and clustering of α3 and α5 integrins into host membrane rafts was also observed in the presence of this fungus (Barros et al., 2016).

Integrins are heterodimeric transmembrane glycoproteins consisting of α and β subunits. In mammals, 18 α and 8 β integrin subunits non-covalently dimerize to form 24 different receptors. Integrins, a major class of receptors involved in cell adhesion to other cells or to extracellular matrix, are able to bind to a wide variety of ligands, including adhesive proteins present on the surface of pathogens. Therefore, in this manner, several pathogens hijack host cell signaling to invade and survive in the host, leading to the establishment of an infection (Hauck et al., 2012).

Integrins may be recruited and clustered into membrane rafts (Leitinger and Hogg, 2002; Wang et al., 2013). These cell membrane structures are dynamic nanoscale domains enriched in sterols, sphingolipids, and specific proteins. Upon stimulation, membrane rafts coalesce to form larger platforms, compartmentalizing and activating cell signaling (Simons and Sampaio, 2011). Some pathogens, such as *Listeria monocytogenes*, *Toxoplasma gondii*, and herpes simplex virus, recruit membrane rafts for host cell invasion (Seveau et al., 2004; Gianni et al., 2010; Cruz et al., 2014). Previously, we verified that *P. brasiliensis* is able to recruit these cell domains for host cell adhesion, and also, cytokine secretion (Maza et al., 2008; Barros et al., 2016).

Engagement of integrins may activate Src-family kinases (SFK), which are non-receptor tyrosine kinases that participate in regulating several cellular events, such as cell growth, division, differentiation and survival (Engen et al., 2008; Ingley, 2008; Okada, 2012). In response to pathogens or other stimuli, some studies have also shown SFK involvement in cytokine secretion by epithelial cells (Ren et al., 2005; Kannan et al., 2006; Lin et al., 2006; Bentley et al., 2007; Eucker et al., 2014). For example, Eucker et al. (2014) recently demonstrated that *Campylobacter jejuni* promotes IL-8 secretion by the INT 407 human intestinal epithelial cells, and that this is triggered in response to engagement of β1 integrins and activation of focal adhesion kinase (FAK), paxillin and Src. Our group previously observed that *P. brasiliensis* also induces SFK activation and recruitment of these signaling molecules to A549 cell membrane rafts (Maza et al., 2008), but the participation of integrins in SFK activation and the role of these kinases in *P. brasiliensis* epithelial cell infection were not determined yet.

Regarding host innate immunity and infection mechanisms by human pathogenic fungi, such as *P. brasiliensis* or *H. capsulatum*, most of the studies were performed with cells of the myeloid lineage, which include macrophages, neutrophils and dendritic cells. However, since the first reports describing the secretion of cytokines by epithelial cells in the 1990s (Stadnyk, 1994), various research groups have demonstrated the importance of these cells in modulating the host immune system. Cleaver et al. (2014), for example, recently showed that mice that inhaled Toll-like receptor (TLR) agonists were protected against lethal pneumonia. This protection persisted even after the reduction or depletion of neutrophils, alveolar macrophages, dendritic cells, mature lymphocytes, or natural killer cells. Moreover, airway epithelial cells treated with TLR agonists were able to kill pathogenic bacteria. Thus, the authors concluded that lung epithelial cells are important for pulmonary antimicrobial defense, and for patients with impaired leukocyte-mediated immunity, epithelial cells may be critical for the antimicrobial action (Cleaver et al., 2014).

As studies about interaction of epithelial cells with *H. capsulatum* are still incipient, in the present study, we aimed to analyze the secretion of inflammatory cytokines by A549 epithelial cells during infection with *H. capsulatum* yeasts. To the best of our knowledge, this is the first report describing the role of α3 and α5 integrins, SFK activation, and membrane rafts in *H. capsulatum*-inducible cytokine secretion.

## Materials and Methods

### Fungal Growth Conditions

*Histoplasma capsulatum*, strain 496, was kindly provided by Dr. Olga F. Gompertz, Escola Paulista de Medicina, Universidade Federal de São Paulo, São Paulo, Brazil. Yeast forms were grown 5–7 days at 37°C, 100 rpm, in BHI medium (Brain Heart Infusion, Becton, Dickinson and Company, USA) as described elsewhere (Toledo et al., 2001).

*Histoplasma capsulatum* yeasts, grown for 3 days, were washed three times with Dulbecco’s Modified Eagle’s Medium (DMEM; Sigma, USA) and used for interaction assays with A549 cells.

### A549 Cell Culture

Human lung epithelial cell line A549 was grown in DMEM supplemented with 10% fetal bovine serum (FBS; Vitrocell Embriolife, Brazil), 10 mM HEPES, 100 U/ml penicillin, and 100 μg/ml streptomycin (complete DMEM) at 37°C, 5% CO_2_.

### Analysis of Cytokine Levels in Culture Supernatants of A549 Cells during Incubation with *H. capsulatum*

1.8 × 10^4^ A549 cells were cultured in 24-well plates with complete DMEM. After 72 h, A549 cells were maintained overnight in FBS-free DMEM (FBS starvation). Next, A549 cells were incubated with 2.0 × 10^6^ *H. capsulatum* yeasts [multiplicity of infection (MOI) of 8 yeasts per A549 cell] for 5, 16, or 24 h. After incubation with *H. capsulatum*, culture supernatants were collected and centrifuged at 1300 × *g* to remove fungi. IL-6, IL-8, and IL-10 levels in these supernatants were determined using DuoSet^®^ ELISA Kits (R&D Systems), according to manufacturer’s instructions.

In some experiments, after FBS starvation, A549 cells were incubated for 2 h in FBS-free DMEM containing 0.1, 1, or 10 μM PP2 (an inhibitor of SFK activation, Calbiochem, USA), 1 μg/ml filipin (a cholesterol-binding compound that disrupts membrane rafts, Sigma, USA), or 0.025% or 0.05% DMSO (DMSO concentrations used as vehicle for PP2 and filipin, respectively). And then, *H. capsulatum* yeasts were added to the cultures and incubated for 16 h. IL-6 and IL-8 levels in these culture supernatants were determined as described above.

### Analysis of Integrin Expression in A549 Cells during Incubation with *H. capsulatum*

1.0 × 10^5^ A549 cells were cultured in 6-well plates with complete DMEM. After 48 h, A549 cells were incubated overnight in FBS-free DMEM. Next, A549 cells were incubated with 6.5 × 10^6^ *H. capsulatum* yeasts (MOI of 8 yeasts per A549 cell) for different periods of time. After incubation with fungi, A549 cells were washed three times with phosphate buffered saline (PBS, 10 mM sodium phosphate buffer, pH 7.2, containing 150 mM NaCl), harvested with a cell scraper, and lysed with TNE (25 mM Tris buffer, pH 7.5, with 150 mM NaCl, 5 mM EDTA pH 7.5) containing 1% Brij^®^ 98 (Sigma, USA) and a mixture of inhibitors (IMix – 5 mM Na_3_VO_4_, 100 μM leupeptin, 125 μg/ml aprotinin, 1 mM AEBSF, all inhibitors were purchased from Sigma, USA) for 30 min at 4°C. Protein content in samples was measured using a Micro BCA^TM^ Protein Assay Kit (Thermo Scientific, USA) according to manufacturer’s instructions.

Ten micrograms of protein were loaded per well of SDS-PAGE gels, and expression of integrins was evaluated by Western blot.

### Association of A549 Cell Integrins with *H. capsulatum* Yeasts

1.12 × 10^6^ A549 cells were cultured in 150-mm plates with complete DMEM. After 72 h, A549 cells were maintained overnight in FBS-free DMEM. A549 cells were washed three times with PBS, harvested with cell scraper, and lysed with lysis buffer (50 mM Tris buffer, pH 7.2, containing 150 mM NaCl, 1 mM CaCl_2_, 1% Brij^®^ 98 and IMix) for 30 min at 4°C.

After protein quantification of A549 cell lysates, 1 mg protein in 500 μl was incubated with 5.0 × 10^8^ *H. capsulatum* yeasts at 4°C with gentle shaking. As controls, fungi were incubated with A549 cell-free lysis buffer. After overnight incubation, samples were centrifuged, and supernatants containing *H. capsulatum*-unassociated proteins were collected. Fungi (pellet) were washed five times with 500 μl of lysis buffer. After each washing step, the supernatant containing *H. capsulatum*-unassociated proteins was collected (U_Fractions_). Next, *H. capsulatum*-associated proteins (A_Fraction_) were eluted with 60 μl of sample buffer (125 mM Tris-HCl, pH 6.8; 4% sodium dodecyl sulfate; 20% glycerol; 0.05% bromophenol blue), boiled for 5 min, and centrifuged. Then, aliquots of A549 cell lysates, *H. capsulatum*-associated (A_Fraction_) and unassociated (U_Fractions_) proteins, and A549 cell-free lysis buffer, that was incubated with this fungus, were submitted to SDS-PAGE. Integrins and caspase-3 were analyzed by Western blot using antibodies anti-α3 and α5 integrins, and caspase-3.

### Silencing of Integrins in A549 Cells by Small Interfering RNA (siRNA)

2.0 × 10^5^ A549 cells were cultured in 6-well plates with DMEM supplemented with 10% FBS and 10 mM HEPES in the absence of antibiotics. After 24 h, A549 cells were washed three times with DMEM supplemented with 1% FBS, and then, transfected with Lipofectamine^®^ RNAiMAX (Life Technologies, USA) and Silencer^®^ Select Pre-designed α3 or α5 integrin siRNA (#s7543 and #s7549, Life Technologies, USA) at a final concentration of 10 nM. Silencer^®^ Select Negative Control No. 1 siRNA (Life Technologies, USA) was used as negative control. After 24 h, A549 cells were washed three times with DMEM, and then, incubated with 6.5 × 10^6^ *H. capsulatum* yeasts (MOI of 8 yeasts per A549 cell). After 16 h, culture supernatants were collected for IL-6 and IL-8 analysis. Concomitantly, A549 cells were washed three times with PBS, harvested with a cell scraper, and lysed with TNE containing 1% Brij^®^ 98 and IMix for 30 min at 4°C. Silencing of α3 and α5 integrins was analyzed by Western blot.

### Analysis of SFK Activation during the Interaction of A549 Cells with *H. capsulatum*

1.0 × 10^5^ A549 cells were cultured in 6-well plates with complete DMEM. After 48 h, A549 cells were maintained overnight in FBS-free DMEM to decrease basal phosphorylation of SFK. Next, A549 cells were incubated with 6.5 × 10^6^ *H. capsulatum* yeasts (MOI of 8 yeasts per A549 cell) for 15, 30, 60, 120, or 180 min. After incubation with fungi, A549 cells were washed three times with PBS containing 1 mM Na_3_VO_4_ (PBS/SV), harvested with a cell scraper, and lysed with TNE containing 1% Brij^®^ 98 and IMix for 30 min at 4°C.

In some experiments, A549 cells were transfected with α3 or α5 integrin siRNA, maintained overnight in FBS-free DMEM, and then, incubated with *H. capsulatum* yeasts for 3 h. After incubation with fungi, A549 cells were washed, harvested, and lysed as described above.

Twenty micrograms of protein were loaded per well of SDS-PAGE gels, and SFK activation was evaluated by Western blot.

### Detergent-Resistant Membrane Isolation

1.12 × 10^6^ A549 cells were cultured in 150-mm plates with complete DMEM. After 72 h, A549 cells were maintained overnight in FBS-free DMEM. Next, A549 cells were incubated with 1.5 × 10^8^ *H. capsulatum* yeasts (MOI of 8 yeasts per A549 cell). After 3 h, A549 cells were washed three times with PBS containing 1 mM Na_3_VO_4_ (PBS/SV), and detergent-resistant membranes (DRMs) were isolated as previously described (Maza et al., 2008). Briefly, harvested cells were lysed with TNE containing 1% Brij^®^ 98 and IMix for 30 min at 4°C. Next, after Dounce-homogenization, the lysate was centrifuged at 1300 × *g*, for 7 min, and the supernatant obtained, termed post-nuclear fraction, was subjected to protein quantification. Same amount of protein was submitted to sucrose gradient, and centrifuged at 260,800 × *g* at 4°C, using the SW 41 Ti rotor Beckman Coulter. After 16 h, 12 fractions of 1 ml each were collected and numbered from top to bottom. Aliquots of DRM fractions (fractions 4–6) and non-DRM fractions (fractions 10–12) were submitted to SDS-PAGE and analyzed by Western blot.

In some experiments, the membrane cholesterol of the cell homogenate was removed as described elsewhere (Maza et al., 2008). Briefly, A549 cells were incubated with *H. capsulatum* yeasts for 3 h then washed, harvested, and centrifuged, producing a cell pellet that was subsequently incubated with 10 mM methyl-β-cyclodextrin (MβCD) in TNE containing IMix for 30 min at 37°C with gentle shaking. Control experiments were performed in the absence of MβCD. Then, Brij^®^ 98 was added to a final concentration of 1%, and after 30 min at 4°C, DRMs were isolated as described above. Aliquots of DRM fractions (fractions 4–6) were submitted to SDS-PAGE, and analyzed by Western blot.

### SDS-PAGE and Western Blot

Aliquots, each containing the same amount of protein, were loaded onto 10% SDS-PAGE gels and then transferred to PVDF membranes. Next, membranes were blocked with 5% non-fat dry milk (Cell Signaling, USA) in TBST (200 mM Tris buffer, pH 8.0, containing 150 mM NaCl and 0.1% Tween^®^ 20) at room temperature. After 1 h, membranes were incubated with 1% BSA in TBST containing the primary antibodies: mouse anti-α3 integrin (1:1000, sc-374242, Santa Cruz, CA, USA), rabbit anti-α5 integrin (1:1000, #4705, Cell Signaling, USA), mouse anti-glyceraldehyde 3-phosphate dehydrogenase (GAPDH; 1:10000, Cat.No.39-8600, Invitrogen, USA), rabbit anti-caspase-3 (1:1000, #9665, Cell Signaling, USA), rabbit anti-Phospho (P)-SFK (Tyr^416^; 1:1000, #2101, Cell Signaling, USA), rabbit anti-cSrc/SFK (1:1000, sc-18, Santa Cruz, CA, USA), or rabbit anti-caveolin-1 (1:2500, sc-894, Santa Cruz, CA, USA). After overnight incubation at 4°C, membranes were incubated for 1 h at room temperature with HRP-conjugated anti-rabbit (1:2000, #7074, Cell Signaling, USA), or anti-mouse antibodies (1:2000, A-10668, Invitrogen, USA) diluted in 1% BSA in TBST. After each step, membranes were washed three times with TBST.

Reactive proteins were detected using SuperSignal^TM^ West Pico Chemiluminescent Substrate (Thermo Scientific, USA) and were documented with a Uvitec Cambridge System (UVITEC, UK). In some experiments, after immunoblotting with anti-P-SFK, membranes were stripped using Restore Western Blot Stripping Buffer (Thermo Scientific, USA), and reprobed with anti-cSrc/SFK (1:1000, sc-18, Santa Cruz, CA, USA). For protein quantification, densitometric analyses were performed using Scion Image (Scion Corporation, USA).

### Cell Viability and Statistical Significance

The viabilities of A549 cell and *H. capsulatum* were measured by MTT (3-[4,5-dimethylthiazol-2-yl]-2,5-diphenyltetrazolium bromide) assay as described previously (Maza et al., 2008).

Briefly, to determine A549 cell viability, cells were incubated in the presence or absence of 0.1, 1, or 10 μM PP2, 1 μg/ml filipin, or 0.025 or 0.05% DMSO for 2 h and then with *H. capsulatum* yeasts for 16 h. After incubation with fungi, A549 cells were washed three times with DMEM without phenol red and incubated using the same medium containing 0.5 mg/ml MTT (Life Technologies, USA) for 2 h. The medium was removed, formazan was solubilized with DMSO, and absorbance was determined at 540 nm with a microplate reader.

To determine fungal viability, after incubation with PP2, filipin or DMSO for 16 h, *H. capsulatum* yeasts were washed three times with DMEM without phenol red and incubated with 0.5 mg/ml MTT as described above.

Statistical significance was evaluated using Student’s *t*-test. *p* < 0.01 or *p* < 0.05 was considered significant.

## Results

### Cytokine Secretion by A549 Cells during Interaction with *H. capsulatum*

**FIGURE 1:**
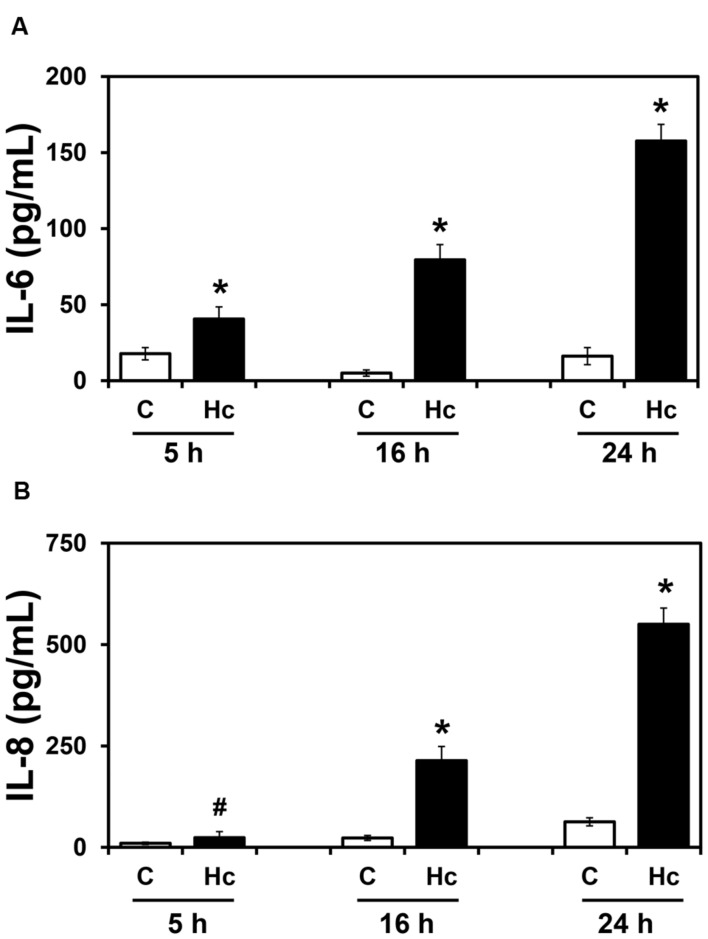
**IL-6 **(A)** and IL-8 **(B)** levels in culture supernatants of A549 cells during interaction with *H. capsulatum***. A549 cells were incubated in the absence (C) or presence (Hc) of *H. capsulatum* yeasts for 5, 16 or 24 h. Culture supernatants were collected, and IL-6 and IL-8 levels were determined by ELISA. Values represent the mean of triplicate experiments ± the standard deviation. ^∗^*p* < 0.01 and #*p* < 0.05 when compared to C of the related time period. Similar results were obtained from three independent experiments.

To verify whether *H. capsulatum* yeasts induce secretion of inflammatory cytokines by epithelial cells, the human lung epithelial cell line A549 was incubated with *H. capsulatum* yeasts for different periods of time (5-24 h), and levels of IL-6, IL-8, and IL-10 in these culture supernatants were determined by ELISA. IL-6 and IL-8 levels significantly increased in a time-dependent manner (**Figure 1**). Regarding IL-6, after 5, 16 and 24 h of A549-*H. capsulatum* interaction, this cytokine levels increased 2.3-, 15.9- and 9.8-fold over basal levels, respectively. Under the same conditions, IL-8 levels increased 2.4-, 9.3- and 8.8-fold for 5, 16 and 24 h time periods, respectively. On the other hand, the anti-inflammatory cytokine IL-10 was undetectable in these culture supernatants (data not shown).

By MTT assay, we verified that cell viability was unaffected when A549 cells were incubated with *H. capsulatum* yeasts for 5–24 h (Supplementary Table 1). Despite this result, as major differences of cytokine levels were observed at 16 h, following experiments were performed within this period.

### Association of α3 and α5 Integrins from Epithelial Cells with *H. capsulatum* Yeasts

First, to determine whether *H. capsulatum* is able to modulate the expression of α3 and α5 integrins, A549 cells were incubated with this fungus for different periods of time (0.5–16 h). By Western blot, it was observed that *H. capsulatum* yeasts were not able to increase the expression levels of these integrins (Supplementary Figure 1).

**FIGURE 2:**
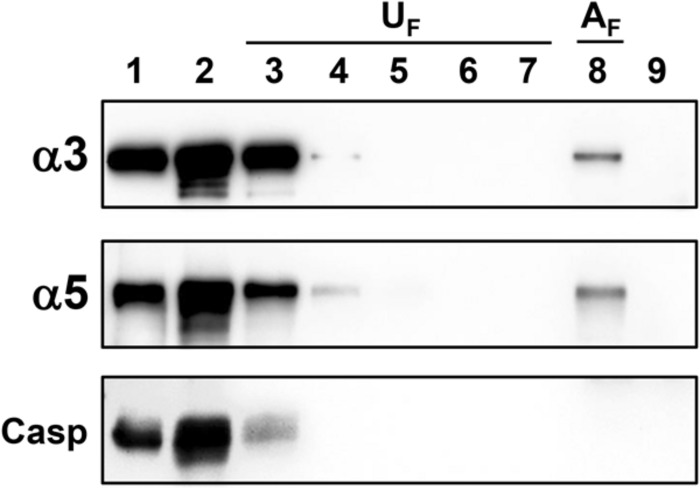
**Association of α3 and α5 integrins from A549 cells with *H. capsulatum* yeasts**. A549 cell lysate was incubated with *H. capsulatum* yeasts overnight at 4°C. Then, samples were centrifuged, and supernatants were collected. Fungi were washed five times to remove unassociated proteins. Next, *H. capsulatum*-associated proteins were eluted with sample buffer. Some fungi were also incubated with A549 cell-free lysis buffer. Lane 1, A549 cell lysate. Lane 2, *H. capsulatum*-unassociated proteins. Lanes 3–7, washing steps (1–5) containing *H. capsulatum*-unassociated proteins (U_F_). Lane 8, associated A549 cell proteins with *H. capsulatum* (A_F_). Lane 9, *H. capsulatum* yeasts incubated with A549 cell-free lysis buffer. Proteins were analyzed by Western blot using antibodies anti-α3 and -α5 integrins. Caspase-3 (Casp) was used as a negative control of association. Blots are representative of two independent experiments.

Next, to evaluate whether α3 or α5 integrin from epithelial cells interacts with *H. capsulatum*, A549 cell lysate was incubated with yeast forms of this fungus. Associated A549 cell proteins with *H. capsulatum* (A_Fraction_ – A_F_) and unassociated proteins from washing steps (U_Fractions_ – U_F_) were analyzed by Western blot. α3 and α5 integrins from A549 cells, associated with yeasts, were eluted and recovered in A_Fraction_ (**Figure 2**, lane 8). As expected, caspase-3 (a cytoplasmatic protein of A549 cell) was not recovered in A_Fraction_ (**Figure 2**, lane 8), indicating that there is no interaction between this cytoplasmatic protein and *H. capsulatum*. In addition, we verified that unassociated proteins were removed efficiently by washing steps (**Figure 2**, lanes 3–7, U_Fractions_). To demonstrate that detected proteins by Western blot were derived from A549 cells, and not from *H. capsulatum*, fungi were also incubated with A549 cell-free lysis buffer. Incubation with this lysis buffer did not extract *H. capsulatum* proteins that could be recognized by the antibodies anti-α3 and anti-α5 integrins (**Figure 2**, lane 9). Therefore, these results indicate that α3 and α5 integrins from A549 cells were associated with *H. capsulatum* yeasts.

### Involvement of α3 and α5 Integrins in Cytokine Secretion by A549 Cells during Interaction with *H. capsulatum*

**FIGURE 3:**
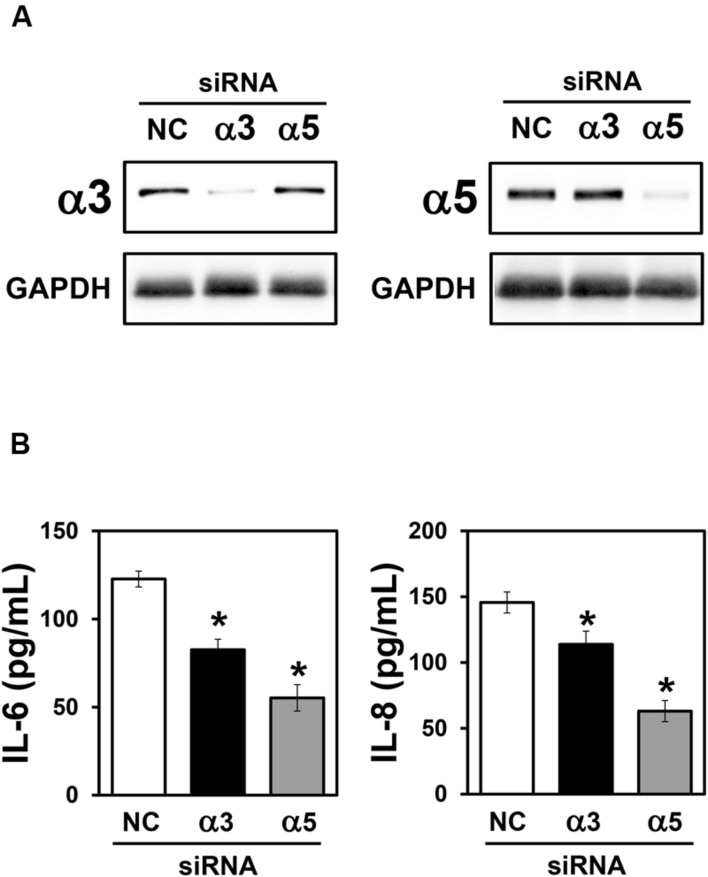
**Effect of α3 or α5 integrin silencing on IL-6 and IL-8 secretion by A549 cells during interaction with *H. capsulatum.*** A549 cells were transfected with α3 or α5 integrin-directed siRNA or with Negative Control (NC) siRNA for 24 h, and then, incubated with *H. capsulatum* yeasts for 16 h. After incubation with fungi, culture supernatants were collected for determination of IL-6 and IL-8 levels, and A549 cells were harvested, lysed, and analyzed by Western blot. **(A)** Silencing of α3 and α5 integrins was confirmed by Western blot. GAPDH was used as protein loading control. Blots are representative of three independent experiments. **(B)** IL-6 and IL-8 levels in culture supernatants were determined by ELISA. Values represent the mean of triplicate experiments ± the standard deviation. ^∗^*p* < 0.01 when compared to NC siRNA. Similar results were obtained from three independent experiments.

Integrins are able to modulate cytokine secretion in various cell types, including epithelial cells (Lubin et al., 2003; Schmid et al., 2004; Gianni and Campadelli-Fiume, 2014). Therefore, to determine the importance of α3 and α5 integrins on IL-6 and IL-8 secretion by A549 cells, during interaction with *H. capsulatum*, silencing of these integrins was performed by using small interfering RNA (siRNA). First, by Western blot (**Figure 3A**) and densitometric analysis (data not shown), we verified that α3 and α5 integrin-directed siRNAs reduced the expression of these receptors by 89 and 87%, respectively, when compared to A549 cells transfected with negative control siRNA.

Next, after incubation with *H. capsulatum* yeasts, we analyzed IL-6 and IL-8 levels in culture supernatants of A549 cells transfected with negative control, α3 or α5 integrin-directed siRNA. By ELISA, it was verified that α3 integrin-directed siRNA was able to reduce significantly 32.7% of IL-6 and 21.9% of IL-8 levels when compared to A549 cells transfected with negative control siRNA (**Figure 3B**). Reduction of IL-6 and IL-8 levels was even more pronounced when α5 integrin-directed siRNA was used (55.0% for IL-6 and 56.7% for IL-8; **Figure 3B**). It was also verified that, in cultures of A549 cells transfected with both integrins (α3 and α5)-directed siRNAs, the decrease of IL-6 and IL-8 levels was the same as observed for A549 cells transfected only with α5-directed integrin siRNA (data not shown). Taken together, these results indicate that α3 and α5 integrins are involved in IL-6 and IL-8 secretion during A549-*H. capsulatum* interaction.

### Activation of Src-Family Kinases (SFK) in A549 Cells during Interaction with *H. capsulatum*

**FIGURE 4:**
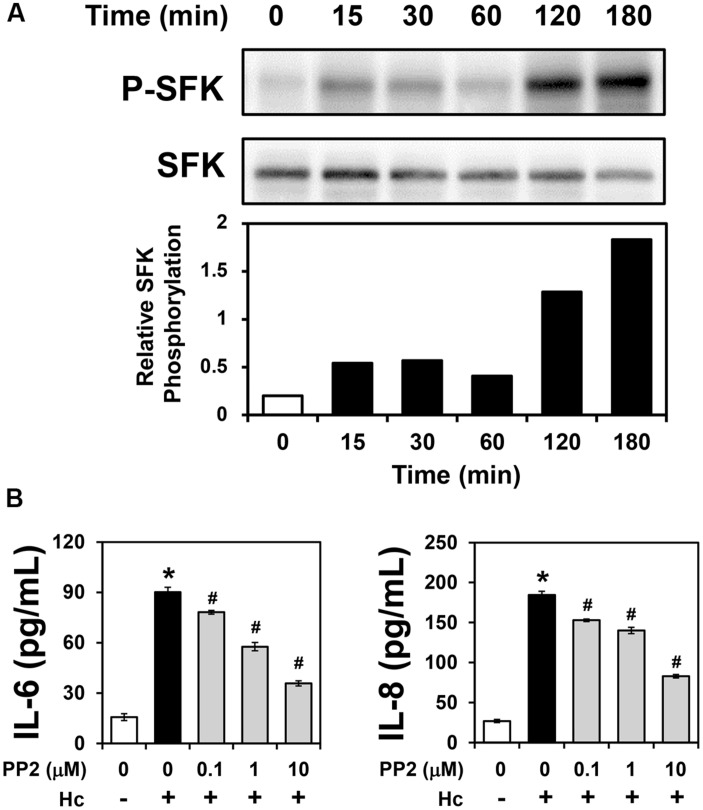
**Activation of SFKs in A549 cells during interaction with *H. capsulatum*, and effect of PP2 on IL-6 and IL-8 secretion by A549 cells during interaction with *H. capsulatum.* (A)** A549 cells were incubated with *H. capsulatum* yeasts for 15, 30, 60, 120 or 180 min, and then P-SFK (Tyr^416^) was analyzed by Western blot. SFK was used as protein loading control. Relative SFK phosphorylation was determined by densitometric analysis of bands obtained by Western blot, and values represent the ratio of the intensity of P-SFK band divided by the corresponding intensity of SFK band. Blots are representative of three independent experiments. **(B)** A549 cells were incubated for 2 h in the absence or presence of 0.1, 1, or 10 μM PP2 (an inhibitor of SFK activation), and then, with *H. capsulatum* yeasts (Hc) for 16 h. After incubation with fungi, culture supernatants were collected, and IL-6 and IL-8 levels were determined by ELISA. To analyze basal cytokine levels, A549 cells were incubated in the absence of PP2 and *H. capsulatum* (PP2 0/Hc -). Values represent the mean of triplicate experiments ± the standard deviation. ^∗^*p* < 0.01 when compared to A549 cells incubated in the absence of PP2 and *H. capsulatum* (PP2 0/Hc -). #*p* < 0.01 when compared to A549 cells incubated with *H. capsulatum* in the absence of PP2 (PP2 0/Hc +). Similar results were obtained from three independent experiments.

Some pathogens exploit integrins for host cell adhesion and invasion, triggering activation of several signaling molecules, including downstream tyrosine kinases such as SFKs (Scibelli et al., 2007; Ulanova et al., 2009; Hauck et al., 2012). Therefore, in order to verify whether A549-*H. capsulatum* interaction induces SFK activation, A549 cells were first incubated with *H. capsulatum* yeasts for different periods of time (15–180 min), and then, levels of SFK phosphorylated at Tyr^416^ (P-SFK) were analyzed by Western blot. As shown in **Figure 4A**, *H. capsulatum* induced SFK activation as early as 15 min, increasing up to 9.1-fold over basal levels after 3 h of A549 cell-*H. capsulatum* interaction.

### Involvement of SFK Activation in Cytokine Secretion by A549 Cells during Interaction with *H. capsulatum*

PP2 (an inhibitor of SFK activation) was used to determine the role of SFK activation in cytokine secretion during A549-*H. capsulatum* interaction. By ELISA, cytokine levels were evaluated in A549 cell-*H. capsulatum* cultures, it was verified that PP2 decreased in a dose-dependent manner IL-6 and IL-8 levels up to 60.3 and 55.1%, respectively (**Figure 4B**). The IC_50_ values of PP2 for IL-6 and IL-8 levels were 3.9 and 7.6 μM, respectively. Taken together, these results indicate that *H. capsulatum* promotes IL-6 and IL-8 secretion in A549 cells in an SFK activation-dependent manner.

A549 cell and fungal viabilities were verified by MTT assay in these experiments. More than 97.0% of A549 cells were viable in the presence of *H. capsulatum* yeasts and PP2, and no morphological changes were observed (Supplementary Table 2). *H. capsulatum* yeasts were viable in the presence of different concentrations of PP2 (Supplementary Table 3).

### Role of α3 and α5 Integrins in SFK Activation in A549 Cells during Interaction with *H. capsulatum*

**FIGURE 5:**
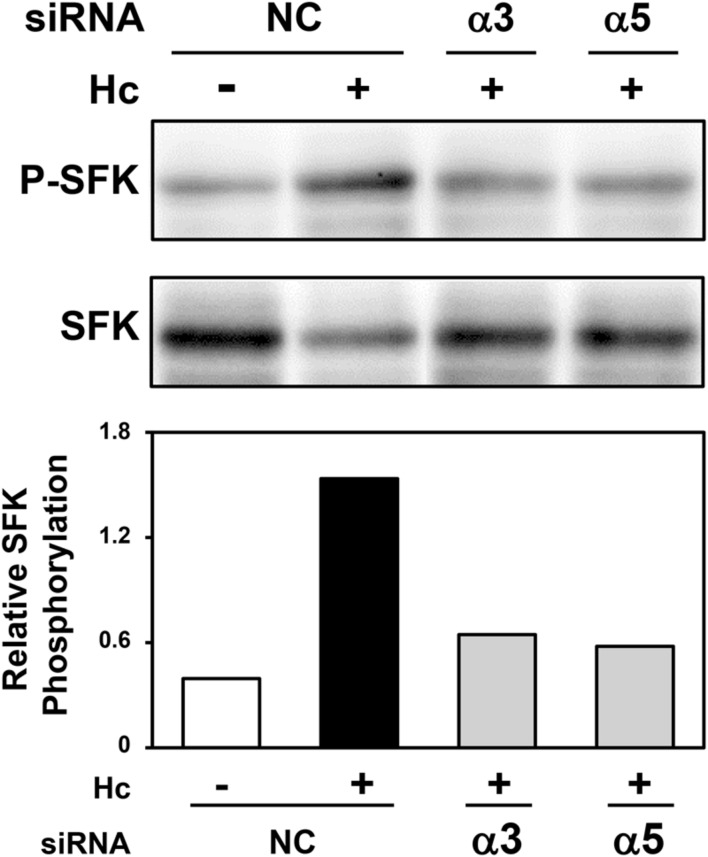
**Effect of α3 or α5 integrin silencing on SFK activation in A549 cells during interaction with *H. capsulatum.*** A549 cells were transfected with α3 or α5 integrin-directed siRNA or with Negative Control (NC) siRNA for 24 h, and then, incubated with *H. capsulatum* yeasts for 3 h. After incubation with fungi, A549 cells were harvested, lysed, and then P-SFK (Tyr^416^) was analyzed by Western blot. SFK was used as protein loading control. Relative SFK phosphorylation was determined by densitometric analysis of the bands obtained by Western blot, and values represent the ratio of the intensity of P-SFK band divided by the corresponding intensity of SFK band.

A549 cells were transfected with α3 or α5 integrin-directed siRNA, and then, incubated with *H. capsulatum* in order to evaluate the importance of these cell receptors on SFK activation. By Western blot and densitometric analyses, it was verified that α3 integrin-directed siRNA reduced SFK activation by 57% up to 64% when compared to A549 cells transfected with negative control siRNA, and incubated with fungi. In addition, SFK activation was decreased by 42% up to 62% when A549 cells were transfected with α5 integrin-directed siRNA (**Figure 5** and Supplementary Figure 2). These results indicate that the interaction between *H. capsulatum* and α3 and α5 integrins is important for SFK activation in A549 epithelial cells. Silencing of these integrins under the same culture conditions was confirmed by Western blot (data not shown).

### Localization of α3 and α5 Integrins and SFKs in Epithelial Cell Membranes Rafts during A549 Cell-*H. capsulatum* Interaction

As previous reports have demonstrated that integrins and/or SFKs may be recruited to membrane rafts under a particular stimulation (Leitinger and Hogg, 2002; Maza et al., 2008; Wang et al., 2013), we investigated whether *H. capsulatum* promotes the recruitment of these proteins to A549 cell membrane rafts. To study this event, DRMs, which contain membrane rafts, were isolated by sucrose gradient/ultracentrifugation method.

**FIGURE 6:**
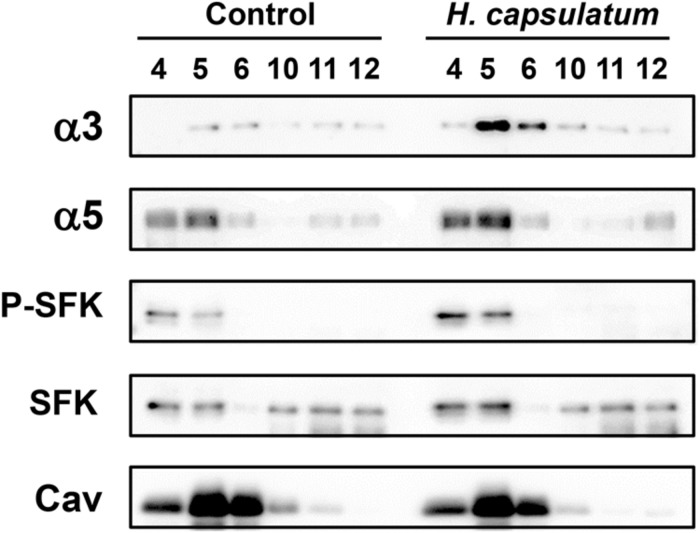
**Localization of SFKs, α3 and α5 integrins in A549 cell DRMs during interaction with *H. capsulatum***. A549 cells were incubated in the absence (Control) or presence of *H. capsulatum* yeasts. After 3 h, A549 cell DRMs were isolated by using a sucrose gradient and ultracentrifugation. Aliquots of DRM fractions (4–6) and non-DRM fractions (10–12) were submitted to SDS-PAGE. α3 and α5 integrins, P-SFK (Tyr^416^), SFK and caveolin-1 were analyzed by Western blot. Caveolin-1 (Cav) was used as a marker of DRM isolation efficiency. Blots are representative of three independent experiments.

After A549-*H. capsulatum* interaction, it was verified the dislodgment of α3 and α5 integrins from non-DRMs (fractions 10–12) to DRMs (fractions 4–6; **Figure 6**). Regarding SFK activation, incubation of epithelial cells with this fungus promoted an increase of P-SFK (Tyr^416^) levels into DRMs (fractions 4–6; **Figure 6**). As expected, caveolin-1, a marker for DRM isolation efficiency, was observed mostly in A549 cell DRMs (fractions 4–6) of both conditions, i.e., A549 cells incubated in the absence (Control) or presence of *H. capsulatum* (**Figure 6**).

**FIGURE 7:**
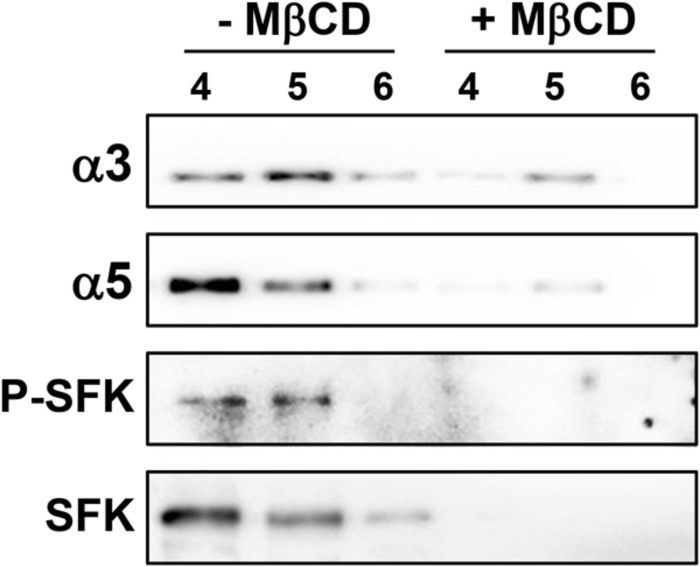
**Effect of membrane raft disruption on the localization of SFKs, α3 and α5 integrins in A549 cell DRMs during interaction with *H. capsulatum***. After incubation with *H. capsulatum* yeasts for 3 h, A549 cells were harvested, lysed and membrane cholesterol of cell homogenate was removed by methyl-β-cyclodextrin (+ MβCD). Control in the absence of MβCD (**-** MβCD) was also performed. Then, A549 cell DRMs were isolated by using a sucrose gradient and ultracentrifugation. Aliquots of DRM fractions (4–6) were submitted to SDS-PAGE. α3 and α5 integrins, P-SFK (Tyr^416^) and SFK were analyzed by Western blot. Blots are representative of two independent experiments.

Membrane rafts are enriched in cholesterol, therefore cholesterol dependence in the recruitment of integrins and SFK to DRMs was analyzed. For this, cholesterol of A549 cell homogenate was depleted with methyl-β-cyclodextrin (MβCD). Treatment with MβCD reduced the levels of α3 and α5 integrins, P-SFK and SFK in DRMs (fractions 4–6; **Figure 7**). Taken together, these results indicate that *H. capsulatum* induces cholesterol-dependent recruitment of these proteins to membrane rafts in A549 cells.

### Effect of Membrane Raft Disruption on IL-6 and IL-8 Secretion by A549 Cells during Interaction with *H. capsulatum*

**FIGURE 8:**
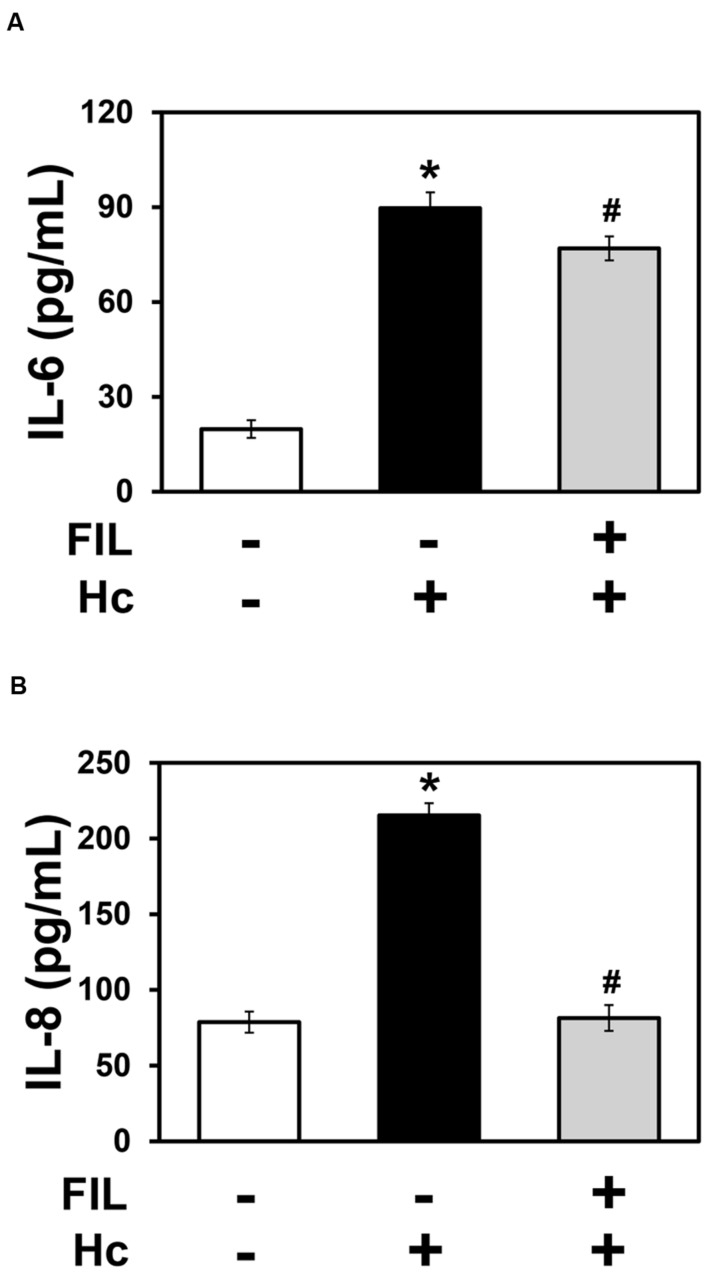
**Effect of filipin on IL-6 and IL-8 secretion by A549 cells during interaction with *H. capsulatum***. A549 cells were incubated in the absence or presence of 1 μg/ml filipin (FIL, a cholesterol-binding compound that disrupts membrane rafts). After 2 h, *H. capsulatum* yeasts (Hc) were added to these cultures. After 16 h, culture supernatants were collected, and IL-6 **(A)** and IL-8 **(B)** levels were determined by ELISA. To analyze basal cytokine levels, A549 cells were incubated in the absence of filipin and *H. capsulatum* (FIL -/Hc -).Values represent the mean of triplicate experiments ± the standard deviation. ^∗^*p* < 0.01 when compared to A549 cells incubated in the absence of filipin and *H. capsulatum* (FIL -/Hc -). #*p* < 0.01 when compared to A549 cells incubated with *H. capsulatum*, in the absence of filipin (FIL -/Hc +). Similar results were obtained from three independent experiments.

The cholesterol-binding compound filipin disrupts membrane rafts, and it was used to evaluate the importance of these domains on *H. capsulatum*-inducible cytokine secretion by A549 cells. By ELISA, it was verified that 1 μg/ml filipin significantly decreased IL-6 and IL-8 levels by 14 and 62%, respectively, when compared to A549 cell cultures incubated with fungi, in the absence of filipin (**Figure 8**). This result indicates, especially for IL-8, that membrane raft recruitment is important for cytokine secretion by A549 epithelial cells during interaction with *H. capsulatum.*

A549 cell and fungal viability was verified by MTT assay. A549 cells were viable in the presence of *H. capsulatum* yeasts and filipin, and no morphological changes were observed (Supplementary Table 4). *H. capsulatum* yeasts were also viable in the presence of filipin (Supplementary Table 5).

## Discussion

Most of the studies about the interaction between pathogenic fungi and host cells are performed using macrophages, dendritic cells, or neutrophils. However, over the last two decades, several groups have demonstrated the ability of epithelial cells to produce inflammatory mediators. In this manner, studies about the epithelial cell mechanisms involved in pathogen-inducible cytokine secretion are important for understanding the role of these cells in the host’s innate immune defense. To the best of our knowledge, this is the first report showing that *H. capsulatum* yeasts are able to promote cytokine release by pulmonary epithelial cells and, more importantly, this event is dependent on α3 and α5 integrins, SFK activation, and membrane rafts clustering.

In this work, it was demonstrated that *H. capsulatum* yeasts promote secretion of the inflammatory cytokines IL-6 and IL-8 by A549 epithelial cells, but not the anti-inflammatory cytokine IL-10. This result was expected because we recently verified that the fungal pathogen *P. brasiliensis* also promotes IL-6 and IL-8 secretion by A549 cells (Maza et al., 2012). Furthermore, other fungi induce cytokine secretion during epithelial cell–fungus interaction. *Aspergillus fumigatus* and *Cryptococcus neoformans*, for example, induce IL-8 secretion by human bronchial epithelial cells (BEAS-2B), while *Candida albicans* promotes IL-6 secretion by human oral epithelial cells (TR146), but not IL-8 (Balloy et al., 2008; Guillot et al., 2008; Moyes et al., 2011), demonstrating that the cytokine profile depends on the pathogen and epithelial cell type studied.

Several groups have demonstrated that host cell integrins are exploited by different pathogens. These microorganisms express molecules that bind directly to integrins, or to extracellular matrix proteins which then associate with integrins. Both ways lead pathogens to manipulate host signaling pathways and subvert cell processes in order to survive and proliferate (Scibelli et al., 2007). Furthermore, pathogen adhesion to host cell surface is essential for establishing infection, and several microorganisms are able to adhere to integrins. In addition, integrins are able to modulate cytokine secretion in different cell types, including epithelial cells (Lubin et al., 2003; Schmid et al., 2004; Gianni and Campadelli-Fiume, 2014).

In this work, it was verified whether integrins are involved in the secretion of IL-6 and IL-8, promoted by *H. capsulatum*, in A549 cells. By siRNA, silencing of α3 and α5 integrins led to IL-6 and IL-8 level reduction in A549-*H. capsulatum* cultures. Therefore, some of the mechanisms by which these integrins induce this cytokine secretion were investigated. First, we verified that *H. capsulatum* interacts with α3 and α5 integrins in A549 cells, because these integrins, present in A549 cell lysates, associated with *H. capsulatum* yeasts. Despite this result, at the moment, we are not certain whether *H. capsulatum* interacts with α3 and α5 integrins directly, or indirectly, by binding to an extracellular matrix ligand. There is a high probability that this interaction is indirect, because *H. capsulatum* yeasts are able to bind to murine laminin that is recognized by α3β1 integrin (McMahon et al., 1995; Kikkawa et al., 1998; Tagliari et al., 2012). This hypothesis is under current investigation in our laboratory.

In addition, pathogens interact with integrins and may lead to cytokine secretion by several mechanisms. For example, the adhesin A of the bacterium *Yersinia enterocolitica* (YadA) mediates adhesion and invasion of HeLa cells and promotes IL-8 secretion by engaging Rho GTPases, MAPKs and NF-κB activation. All these events are dependent on β1 integrin, because blocking antibodies against this integrin reduced IL-8 production and host cell adhesion (Schmid et al., 2004). The periodontopathogen *Treponema denticola* (Td92) is another example that interacts with integrins. Interaction of a surface protein of this bacterium with α5β1 integrin promotes NLRP3 inflammasome activation, IL-1β secretion, and NF-κB signaling pathway (Jun et al., 2012).

Besides integrins, membrane rafts and SFKs may also be involved in cytokine secretion promoted by different pathogens. For example, Cheon et al. (2008) and Im et al. (2009) showed that treatment of A549 cells with nystatin reduces IL-8 secretion promoted by bacterial flagellin and peptidoglycan, indicating the importance of membrane rafts on this cytokine secretion. Regarding SFKs, Kannan et al. (2006) demonstrated that *Pseudomonas aeruginosa* infection promotes Lyn (a member of SFK) activation in A549 cells. In addition, the authors showed that Lyn is involved in cytokine secretion, because PP2 reduced IL-1β levels in A549-*P. aeruginosa* supernatants. In this work, using different approaches, we show that *H. capsulatum* yeasts lead to IL-6 and IL-8 secretion by associating with α3 and α5 integrin, recruiting these integrins to A549 cell membrane rafts, and then activating SFKs.

Comparing the results of our previous work with *P. brasiliensis* (Barros et al., 2016), and those obtained with *H. capsulatum*, we observed some differences in the importance of α3 and α5 integrins in IL-6 and IL-8 secretion by A549 cells. First, although both fungi are able to interact with α3 and α5 integrins, *H. capsulatum* infection did not alter these integrin levels in A549 cells, while *P. brasiliensis* was able to promote an increase of α3 and α5 integrins. Regarding integrin involvement in cytokine secretion, major differences were seen when A549 cells were transfected with α3 integrin siRNA and IL-8 levels were measured. α3 integrin seems to be critical for IL-8 secretion promoted by *P. brasiliensis*, but not by *H. capsulatum*. Therefore, together, these results indicate that each fungal pathogen interacts differently with these epithelial cells, promoting cytokine secretion by several mechanisms.

In addition, besides integrins, several receptors may be involved in cytokine secretion. Some studies have shown the cooperation between integrins and TLRs (Marre et al., 2010; Gianni and Campadelli-Fiume, 2014). For example, Lerman et al. (2014) related the importance of α3β1 integrin expression on TLR-induced cytokine production by neutrophils during sepsis. The authors showed that integrin deletion reduced IL-6 and IL-10 secretion by neutrophils stimulated with Pam_3_CSK_4_ (a TLR2/1 specific stimulus), indicating that α3β1 integrin cooperates with TLR2-induced cytokine responses. Therefore, the involvement of TLRs and other receptors in integrin-mediated cytokine secretion during interaction with pathogenic fungi will also be studied in our laboratory.

## Author Contributions

PM and ES designed the project and experiments, analyzed the data and wrote the manuscript. PM performed all the experiments.

## Conflict of Interest Statement

The authors declare that the research was conducted in the absence of any commercial or financial relationships that could be construed as a potential conflict of interest.
