# Supplementary material for: Histoplasma capsulatum-Induced Cytokine Secretion in Lung Epithelial Cells Is Dependent on Host Integrins, Src-Family Kinase Activation, and Membrane Raft Recruitment
Source: Front Microbiol. 2016 Apr 22;7:580. doi: 10.3389/fmicb.2016.00580 (PMC4840283; doi:10.3389/fmicb.2016.00580)
Supplement: Supplementary file 1 [file Table_1.PDF]

Supplementary Table 1. A549 cell viability in presence of *H. capsulatum* yeasts

Time (h)	Group	Mean ± standard deviation	*p* value
5	C	3.428 ± 0.010	
	Hc	3.409 ± 0.108	0.731
16	C	3.327 ± 0.230	
	Hc	3.222 ± 0.015	0.395
24	C	3.302 ± 0.135	
	Hc	3.284 ± 0.074	0.827

A549 cell viability was measured by MTT assay. A549 cells were incubated in the absence (C) or presence (Hc) of *H. capsulatum* yeasts for 5, 16, or 24 h. After incubation with fungi, A549 cells were washed and incubated with 0.5 mg/ml MTT for 2 h. Formazan was solubilized with DMSO, and absorbance was determined at 540 nm. Values represent means ± standard deviations and *p* when compared with C for the same time period.
