# Supplementary material for: Histoplasma capsulatum-Induced Cytokine Secretion in Lung Epithelial Cells Is Dependent on Host Integrins, Src-Family Kinase Activation, and Membrane Raft Recruitment
Source: Front Microbiol. 2016 Apr 22;7:580. doi: 10.3389/fmicb.2016.00580 (PMC4840283; doi:10.3389/fmicb.2016.00580)
Supplement: Supplementary file 2 [file Table_2.PDF]

Supplementary Table 2. A549 cell viability in presence of PP2 and *H. capsulatum* yeasts

Group	Mean ± standard deviation	*p* value
C	3.263 ± 0.116	
Hc	3.267 ± 0.033	0.956
0.1 μM PP2 + Hc	3.215 ± 0.129	0.600
1 μM PP2 + Hc	3.259 ± 0.047	0.948
10 μM PP2 + Hc	3.170 ± 0.090	0.251

A549 cell viability was measured by MTT assay. A549 cells were incubated with 0.1, 1, or 10 μM PP2 or 0.025% DMSO for 2 h, and then, in the absence (C) or presence (Hc) of *H. capsulatum* yeasts for 16 h. After incubation with fungi, A549 cells were washed and incubated with 0.5 mg/ml MTT for 2 h. Formazan was solubilized with DMSO, and absorbance was determined at 540 nm. Values represent means ± standard deviations and *p* when compared to A549 cells incubated in the absence of PP2 and *H. capsulatum* (C).
