# Supplementary material for: Histoplasma capsulatum-Induced Cytokine Secretion in Lung Epithelial Cells Is Dependent on Host Integrins, Src-Family Kinase Activation, and Membrane Raft Recruitment
Source: Front Microbiol. 2016 Apr 22;7:580. doi: 10.3389/fmicb.2016.00580 (PMC4840283; doi:10.3389/fmicb.2016.00580)
Supplement: Supplementary file 3 [file Table_3.PDF]

Supplementary Table 3. *H. capsulatum* viability in presence of PP2

Group	Mean ± standard deviation	*p* value
C	3.073 ± 0.232	
0.1 μM PP2	3.215 ± 0.129	0.660
1 μM PP2	3.259 ± 0.047	0.886
10 μM PP2	3.170 ± 0.090	0.424

H. capsulatum viability was measured by MTT assay. After incubation with 0.1, 1, or 10 μM PP2 or 0.025% DMSO (C) for 16 h, *H. capsulatum* yeasts were washed and incubated with 0.5 mg/ml MTT for 2 h. Formazan was solubilized with DMSO, and absorbance was determined at 540 nm. Values represent means ± standard deviations and *p* when compared to *H. capsulatum* yeasts incubated in the absence of PP2 (C).
