# Supplementary material for: Histoplasma capsulatum-Induced Cytokine Secretion in Lung Epithelial Cells Is Dependent on Host Integrins, Src-Family Kinase Activation, and Membrane Raft Recruitment
Source: Front Microbiol. 2016 Apr 22;7:580. doi: 10.3389/fmicb.2016.00580 (PMC4840283; doi:10.3389/fmicb.2016.00580)
Supplement: Supplementary file 5 [file Table_5.PDF]

**Supplementary Table 5. *H. capsulatum* viability in presence of filipin**

| Group | Mean ± standard deviation | *p* value |
|---|---|---|
| C | 2.337 ± 0.031 | |
| 1 μg/mL FIL | 2.365 ± 0.019 | 0.178 |

*H. capsulatum* viability was measured by MTT assay. After incubation with 1 μg/ml filipin (FIL) or 0.05% DMSO (C) for 16 h, *H. capsulatum* yeasts were washed and incubated with 0.5 mg/ml MTT for 2 h. Formazan was solubilized with DMSO, and absorbance was determined at 540 nm. Values represent means ± standard deviations and *p* when compared to *H. capsulatum* yeasts incubated in the absence of filipin (C).
