# Supplementary figures and images for: Histoplasma capsulatum-Induced Cytokine Secretion in Lung Epithelial Cells Is Dependent on Host Integrins, Src-Family Kinase Activation, and Membrane Raft Recruitment
Source: Front Microbiol. 2016 Apr 22;7:580. doi: 10.3389/fmicb.2016.00580 (PMC4840283; doi:10.3389/fmicb.2016.00580)

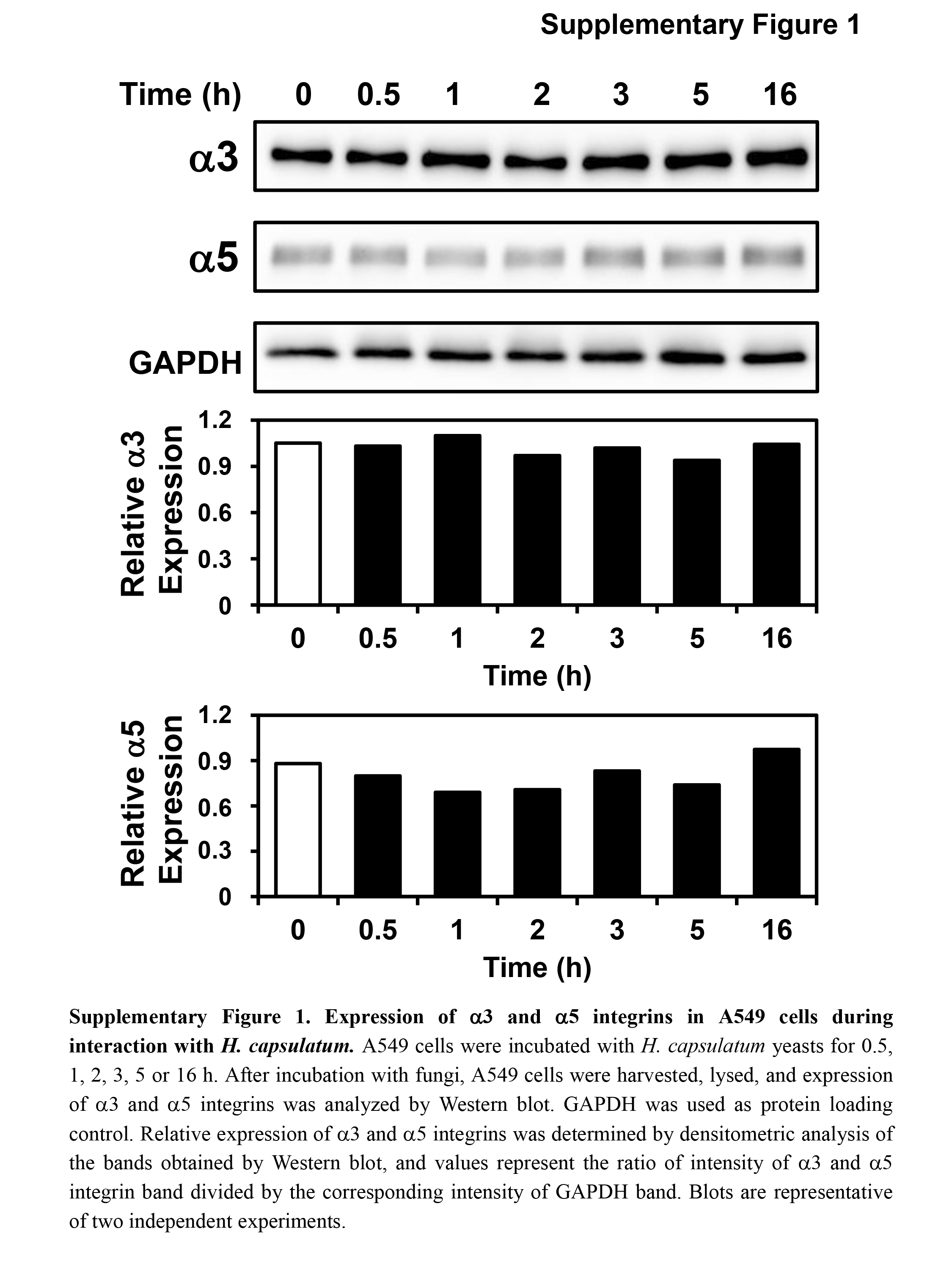

Supplement: Supplementary file 6 [file Image_1.TIF]

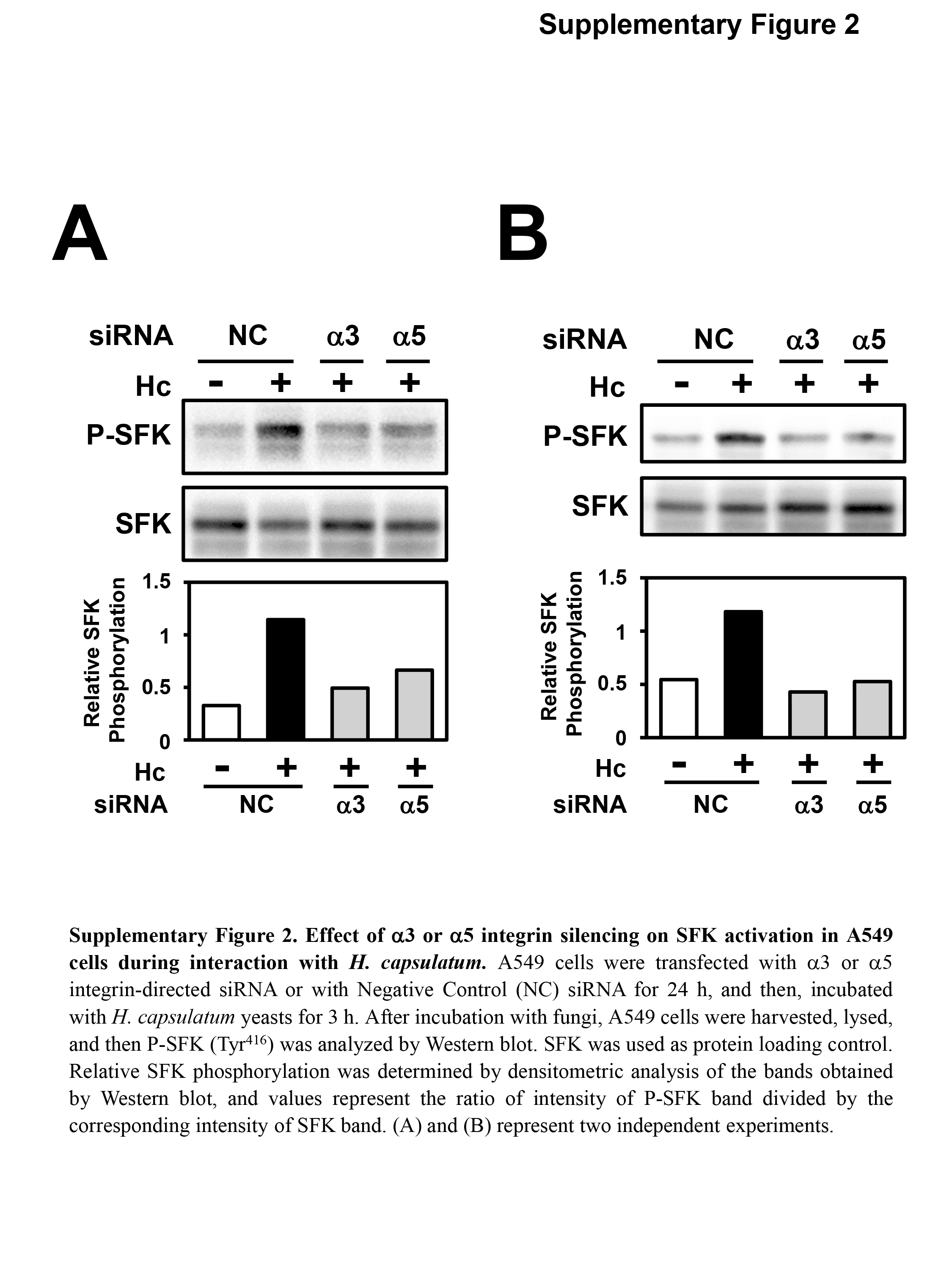

Supplement: Supplementary file 7 [file Image_2.TIF]
